# Measles transmission following the tsunami in a population with a high one-dose vaccination coverage, Tamil Nadu, India 2004–2005

**DOI:** 10.1186/1471-2334-6-143

**Published:** 2006-09-19

**Authors:** Arumugam Mohan, Manoj V Murhekar, Niteen S Wairgkar, Yvan J Hutin, Mohan D Gupte

**Affiliations:** 1 Field Epidemiology Training Programme, National Institute of Epidemiology (ICMR), Chennai, India; 2 Directorate of Public Health and Preventive Medicine, Govt. of Tamil Nadu, Chennai, India; 3 National Institute of Virology (ICMR), Pune, India

## Abstract

**Background:**

On 26 December 2004, a tsunami struck the coast of the state of Tamil Nadu, India, where one-dose measles coverage exceeded 95%. On 29 December, supplemental measles immunization activities targeted children 6 to 60 months of age in affected villages. On 30 December, Cuddalore, a tsunami-affected district in Tamil Nadu reported a cluster of measles cases. We investigated this cluster to estimate the magnitude of the problem and to propose recommendations for control.

**Methods:**

We received notification of WHO-defined measles cases through stimulated passive surveillance. We collected information regarding date of onset, age, sex, vaccination status and residence. We collected samples for IgM antibodies and genotype studies. We modeled the accumulation of susceptible individuals over the time on the basis of vaccination coverage, vaccine efficacy and birth rate.

**Results:**

We identified 101 measles cases and detected IgM antibodies against measles virus in eight of 11 sera. Cases were reported from tsunami-affected (n = 71) and unaffected villages (n = 30) with attack rates of 1.3 and 1.7 per 1000, respectively. 42% of cases in tsunami-affected villages had an onset date within 14 days of the tsunami. The median ages of case-patients in tsunami-affected and un-affected areas were 54 months and 60 months respectively (p = 0.471). 36% of cases from tsunami-affected areas were above 60 months of age. Phylogenetic analyses indicated that the sequences of virus belonged to genotype D8 that circulated in Tamil Nadu.

**Conclusion:**

Measles virus circulated in Cuddalore district following the tsunami, although there was no association between the two events. Transmission despite high one-dose vaccination coverage pointed to the limitations of this vaccination strategy. A second opportunity for measles immunization may help reducing measles mortality and morbidity in such areas. Children from 6 month to 14 years of age must be targeted for supplemental immunization during complex emergencies.

## Background

Measles remains an important cause of childhood mortality, especially in developing countries. In 2000, measles killed 770,000 children worldwide, accounting for nearly half of vaccine preventable deaths [1]. Failure to deliver at least one dose of measles vaccine to all infants remains the primary reason for high measles mortality and morbidity in developing countries [2]. Measles vaccination coverage among infants in Southeast Asia and Africa is still low, ranging between 54–55% in 1999 to 65–67% in 2003 [3]. However, Sri Lanka [4], Latin America [5], Romania [6] and South Korea [7], experienced outbreaks of measles in spite of sustained high coverage with single-dose vaccination strategy. Thus, the 2001–2005 WHO/UNICEF strategic plan for measles mortality reduction and regional elimination recommended achieving high routine vaccination coverage (>90%) in every district and ensuring that all children receive a second opportunity for measles immunization [2].

Population movement and high population densities facilitate transmission of the measles virus. Thus, outbreaks of measles are common among refugees and displaced populations [8-11]. Poor nutritional status, also common in these settings, has been associated with an increased risk of death following measles [11,12]. As a result, measles outbreaks are a major killer during complex emergencies. Immunization of children against measles is considered a highly cost-effective priority among displaced populations housed in camps [13]. WHO and UNICEF recommend vaccinating all children from six months through 14 years of age along with vitamin A supplementation during emergencies [14]. At the minimum, children from six months through 4 years must be vaccinated, while vaccine availability, funding, human resources and local measles epidemiology may influence the choice of the age groups covered [14].

On 26 December 2004, a tsunami struck the coast of the state of Tamil Nadu, India, affecting more than 896,000 individuals and resulting in 7,983 deaths [15]. Affected individuals were provided with shelter in temporary camps. On 28 December 2004, the Field Epidemiology Training Programme of the National Institute of Epidemiology, Chennai, Tamil Nadu, sent three teams to the districts of Cuddalore, Nagapatinam and Kanyakumari that had been affected by the tsunami to assist district health authorities in (1) stimulating the routine surveillance system so that it would meet the needs of the emergency and (2) responding to outbreaks. On 29 December 2004, health authorities initiated supplemental measles immunization targeting children aged six to 60 months in all tsunami-affected villages in Tamil Nadu. On 30 December 2004, the emergency surveillance system set-up by the Indian Field Epidemiology Training Programme detected a cluster of measles in one of the relief camps in the district of Cuddalore. The estimated measles vaccine coverage exceeded 95% since 1988 in Tamil Nadu. (Director of Public Health, Govt. of Tamil Nadu, unpublished data). We investigated this cluster to estimate the magnitude of the problem and to propose recommendations for control.

## Methods

### Descriptive epidemiology

We defined a case of measles as per WHO guidelines as the occurrence of fever with maculo-papular rash and at least one of the following: cough, coryza or conjunctivitis in a resident of the Cuddalore district from 26 December 2004 [16]. We received notification of measles cases through the routine passive surveillance system that we stimulated following tsunami by requesting health care facilities in the district to report occurrences of measles and other diseases of epidemic potential [17]. For each case, we collected information regarding date of onset, age, sex, vaccination status and place of residence. We sorted places of residence to determine whether the case occurred in any of the 58 coastal villages declared as tsunami affected (2005 population = 87,284) by the Government of Tamil Nadu [18]. In the absence of reported major measles outbreak since 1988, we modeled the accumulation of children susceptible to measles since 1988, according to census data, population growth rate, birth rate, administrative vaccination coverage for the state of Tamil Nadu and the expected vaccine efficacy of 85% for measles vaccine administered at 9 months of age [19] according to the method proposed by de Quadros [20]. We calculated the coverage of supplementary measles immunization in tsunami-affected areas using the administrative method by dividing the number of doses of vaccine administered during the campaign by the number of children aged six to sixty months in this area. We applied the proportion of population aged 6–60 months in the district to the size of the affected population to calculate number of children in this age group in tsunami-affected area. The administrative coverage may exceed 100% because of census inaccuracies, population movements and vaccination of children beyond the recommended age group.

### Laboratory confirmation

We collected blood samples from randomly selected case patients with active clinical measles and examined sera for IgM antibodies against measles virus (Behring, Germany). We collected throat swabs from case patients with active clinical measles and performed RT PCR using N gene primers [21]. We performed the sequence alignment and phylogenetic tree analysis using Clustal X (version 1.83) and MEGA (version, 2.1) software and built a phylogenetic tree with standard WHO sequences of each genotype.

### Ethical clearance

We conducted this investigation under the responsibility of the National Institute of Epidemiology, according to the ethical guidelines for biomedical research on human subjects of Indian council of Medical Research. The National Institute of Epidemiology has its own Ethical Committee. However, this investigation was conducted in the context of a public health response to an outbreak and therefore ethical committee review was not indicated.

### Data analysis

We entered and analyzed the data using Epi Info software (Version 3.3). We calculated the attack rates for measles in areas affected and un-affected by the tsunami in Cuddalore district using population data obtained from the district health authorities. As the age distribution was not available at the village level, we applied the age distribution of the district to calculate the age-group specific attack rates in affected and unaffected areas. The median ages of measles cases in tsunami affected and un-affected population was compared using non-parametric test.

## Results

We identified 101 cases that met the WHO case definition for measles. There were no deaths. We collected eleven blood samples from the case-patients for serological testing. Of these, eight were positive for IgM antibodies against measles virus. We took throat swabs from two children with active measles for the isolation of the virus. Phylogenetic analysis indicated that both the sequences of measles virus belonged to genotype D8.

**Figure 1:**
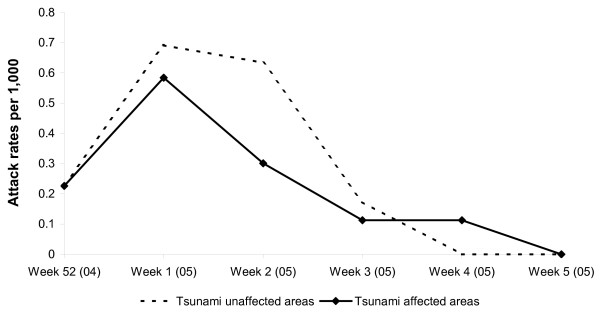
Attack rates of measles by week of onset in tsunami-affected and unaffected areas, Cuddalore district, Tamilnadu, India, December 2004 – January 2005.

Cases were reported with dates of onset between December 2004 and January 2005 (Figure 1) from tsunami-affected and unaffected areas. In both areas, the number of cases reported peaked during the second week following the first case. In tsunami-affected areas, 30 of the 71 cases (42%) occurred within 14 days of the tsunami. 71 measles cases were reported from 20 tsunami-affected villages (2005 population: 53,104) while the remaining 30 cases were from nine unaffected villages (2005 population: 17,357). The attack rate in tsunami-affected areas was lower than in tsunami un-affected areas, although the difference was not statistically significant (p = 0.24). The median ages of case-patients in tsunami-affected and un-affected areas were 54 months (range: 4 months-12 years) and 60 months (range: 6 months-21 years), respectively (p = 0.471). There was no major difference in the age-specific attack rates in tsunami-affected and unaffected areas (Table-1). 26 of the 71 case patients (36.2%) from tsunami-affected areas and 14 of 30 (46.7%) cases in tsunami unaffected areas were above 60 months of age. Among the 101 cases, three (3%) had vaccination records indicating they had received measles vaccine, three (3%) had vaccination records indicated that they had not received measles vaccine and 95 (94%) had no vaccination records.

**Table 1:** Incidence of measles by age and sex in tsunami affected and un-affected villages of Cuddalore district, Tamil Nadu, December 2004- January 2005

	Tsunami affected areas			Tsunami un-affected areas		
Age group (years)	Number of cases (%)	Population	Incidence per 1000	Number of cases (%)	Population	Incidence per 1000

0–4	31 (43.7)	4360	7.1	11 (36.7)	1425	7.7
5–9	32 (45.1)	4786	6.7	16 (53.3)	1564	10.2
10–14	8 (11.3)	5138	1.6	2 (6.7)	1679	1.2
>14	0 (0.0)	38820	0.0	1 (3.3)	12689	0.1

Sex						

Male	32 (45.1)	26720	1.2	16 (53.3)	8734	1.8
Female	39 (54.9)	26384	1.5	14 (46.7)	8623	1.6

Total	71 (100.0)	53104	1.3	30 (100.0)	17357	1.7

The supplementary measles immunization initiated on 29 December 2004 lasted till 9 January 2005 (second week after tsunami). The estimated population of children aged 6 to 60 months in tsunami-affected area was 8803. A total of 10, 319 children were administered measles vaccine (Director of Public Health, Govt. of Tamil Nadu, unpublished data). The coverage for the supplementary campaign calculated using the administrative method was 117.2%. The results of our modeled estimate of population size of susceptible children suggested that between 1988 and 2004, the population of susceptible children in the district was 97,401 exceeding the size of two birth cohorts (Table 2).

**Table 2:** Accumulation of children susceptible to measles in the Cuddalore district, Tamil Nadu, India, 1988 – 2004

Year	Estimated population^#^	Birth cohort	Vaccination coverage*	Vaccine efficacy**	Children susceptible	Cumulated susceptible population
1988	2,076,264	44,432	103.19%	85%	5,459	5,459
1989	2,091,648	46,853	105.50%	85%	4,837	10,296
1990	2,107,146	44,461	101.67%	85%	6,038	16,334
1991	2,122,759	44,153	100.65%	85%	6,378	22,712
1992	2,138,488	44,267	104.57%	85%	4,921	27,633
1993	2,154,333	42,009	102.70%	85%	5,337	32,970
1994	2,170,295	41,670	102.51%	85%	5,362	38,332
1995	2,186,376	44,383	104.92%	85%	4,803	43,135
1996	2,202,576	42,289	101.57%	85%	5,779	48,913
1997	2,218,896	42,603	100.74%	85%	6,124	55,037
1998	2,235,337	42,918	99.28%	85%	6,700	61,737
1999	2,251,900	43,462	101.25%	85%	6,058	67,795
2000	2,268,586	43,784	101.13%	85%	6,148	73,944
2001	2,285,395	44,108	105.18%	85%	4,674	78,617
2002	2,302,329	44,435	102.56%	85%	5,697	84,314
2003	2,319,388	44,764	101.46%	85%	6,159	90,473
2004	2,336,574	45,096	99.57%	85%	6,928	97,401

^#^Census data 2001. Population growth rate of 11.19%

*Vaccination coverage using administrative method

**Vaccination between nine and 12 month of age.

## Discussion

Measles virus circulated between December 2004 and February 2005 in tsunami-affected and un-affected areas of the district of Cuddalore, Tamil Nadu. Transmission occurred despite high one-dose measles vaccine coverage in the state. Active surveillance in tsunami affected areas with early case detection and management might have contributed to the absence of fatality. In addition, supplemental measles immunization targeting children between six and sixty months might have decreased the population of susceptible children in tsunami affected areas which might have limited the spread of this cluster.

The findings of the present investigation suggest that measles transmission in the affected area was unrelated to the tsunami for a number of reasons. First, attack rates were not statistically different in tsunami affected and unaffected areas. Second, the attack rate over time showed a similar pattern in both areas. Third, a large proportion of cases occurred within one incubation period after the occurrence of the tsunami. Fourth, the age distribution of the cases did not differ in tsunami affected and unaffected areas. Finally, the size of shelters in tsunami affected areas in Cuddalore ranged from 115 to 2500 (average: 656)[17]. It was much smaller than the major population concentration usually leading to outbreaks in refugee setting [22].

This cluster of measles occurred despite a high one-dose measles vaccine coverage in Tamil Nadu (Director of Health Services, unpublished data). An independent LQAS vaccine coverage survey conducted in one primary health care center area also confirmed high one-dose coverage in the state [23]. The results of the phylogenetic analysis indicated that both the sequences of measles virus belonged to genotype D8. Genotype D4 and D8 have been isolated from different districts of Tamil Nadu over the last four years from sporadic cases as well as during outbreaks (National Institute of Virology, unpublished data). The absence of importation of a new genotype further supports the hypothesis that a measles virus of that genotype circulates in the area despite high one-dose vaccination coverage. Measles transmission in areas with high one-dose vaccination coverage points to the limitation of the one dose measles vaccination strategy in measles control. Our model suggested that even with a high coverage scenario, the one-dose measles immunization strategy in place in Tamil Nadu allowed an accumulation of a population of susceptible children that exceeded the size of two birth cohort, thereby allowing transmission. Outbreaks of measles may occur when the population of susceptible in a community exceeds one birth cohort [20]. Measles outbreaks in areas with high coverage with a single-dose strategy have been reported in Sri Lanka [4], Latin American countries [5] and in Romania [6]. A second opportunity for measles immunization in areas with high one-dose coverage may provide additional immunity needed for effective measles control [24]. Our model also suggested that children in the district could have benefited from a second opportunity for measles vaccination. To reduce the measles mortality in India, the Government of India developed a strategic plan in 2005 with the objective of (1) reducing the measles mortality by two-thirds by 2010, compared to 2000 estimates and (2) achieving at least 90% coverage with measles vaccine in 80% of the districts of the country by 2009 [25]. In addition, states like Tamil Nadu where one-dose coverage exceeds 90%, the existing network of the national polio surveillance project will be used to collect good quality epidemiological data through active surveillance and outbreak investigations.

During this cluster, the number of cases increased and decreased sharply over time. When measles affects more than one village, transmission is expected to last for number of incubation periods. The decrease of incidence observed in the present investigation is unlikely to be due to surveillance bias. The passive surveillance system continued to be stimulated for a number of weeks following the tsunami and was progressively included as a pilot project in the Integrated Disease Surveillance project (IDSP), the new national surveillance initiative. Supplemental immunization activities that took place at the time of the outbreak might have contributed to the rapid decrease of incidence. These included (1) the post-tsunami immunization campaign that targeted children of 6–60 months between 29 December 2004 and 9 January 2005 in the affected areas and (2) a practice referred to as 'ring immunization' under which children aged 6–60 months of age living within a radius of 5 kilometers of the case of measles. Our investigation however was not able to document any age shift in the cases following the campaign as the outbreak ended rapidly. More generally, we were not able to quantify the impact of supplementary immunization activities on the dynamic of the outbreak.

We did not examine possible missed opportunities for measles immunization in this investigation. Missed opportunities for immunization are important to identify to increase immunization coverage [26]. As a consequence, we were unable to propose specific recommendations to eliminate them. However, the high one-dose measles coverage in the state of Tamil Nadu suggests that addressing the need of a second opportunity for measles vaccination is of higher priority that preventing missed opportunities in this specific setting.

The results of our investigation suggested that measles virus transmission continued despite high one-dose measles vaccine coverage and that the cluster was unrelated to the tsunami. The World Health Organization recommends vaccinating children aged six months to 14 years in refugee and internally displaced persons camps [14] but also mentions that age group to be targeted during such campaigns depends on the local epidemiology of the disease. In Tamil Nadu, the high one-dose coverage may have contributed to the decision to restrict the target age group to children 6 to 60 month of age after the tsunami emergency. However, vaccinating children only up to five years of age might have left some children susceptible to measles as more than 36% of the cases were older than five years of age. The results of our investigation support the recommendation to vaccinate children up to 14 years of age during complex emergencies, even if the one-dose coverage is high. It is also necessary that information, education and communication activities targeting mothers as well as health care workers emphasize the importance of vaccination cards to allow appropriate evaluation of vaccination coverage in Tamil Nadu. In the longer term, the ministry of health of the government of India will be examining how best to address measles as a public health problem given the heterogeneity of the country with respect to measles vaccination coverage. While some states will need to focus on increasing the one-dose vaccination coverage, a number of southern states might be considered for second opportunity of measles vaccination.

## Conclusion

The present cluster of measles was unrelated to accumulation of susceptible population on account of tsunami. Measles transmission despite high one-dose vaccination coverage points towards the need of second opportunity of measles immunization. During complex emergencies, measles immunization should be offered to children from 6 month to 14 years of age.

## Abbreviations

WHO: World Health Organization

UNICEF: United Nations Children's Fund

PCR: Polymerase Chain Reaction

## Conflict of interest

The author(s) declare that they have no competing interests.

## Authors' contributions

AM and MVM were involved in data acquisition, analysis and interpretation of the data, MVM and NSW were involved in drafting the manuscript, NSW carried out laboratory analysis, MDG and YH have made substantial contributions to conception and design of the investigation and revised it critically for intellectual content. All authors read and approved the final manuscript.

## Pre-publication history

The pre-publication history for this paper can be accessed here:


